# Publisher Correction: UvrD helicase–RNA polymerase interactions are governed by UvrD’s carboxy-terminal Tudor domain

**DOI:** 10.1038/s42003-020-01426-x

**Published:** 2020-11-05

**Authors:** Ashish A. Kawale, Björn M. Burmann

**Affiliations:** 1 Wallenberg Centre for Molecular and Translational Medicine, University of Gothenburg, Gothenburg, 40530 Sweden; 2 Department of Chemistry and Molecular Biology, University of Gothenburg, Gothenburg, 40530 Sweden

**Keywords:** Solution-state NMR, DNA-binding proteins

Correction to: *Communications Biology* 10.1038/s42003-020-01332-2, published online 23 October 2020.

In the original published version of the article, the labels to Fig. 1e and Fig. 1f were corrupted. The errors have been corrected in the HTML and PDF versions of the article.

