# Supplementary material for: UvrD helicase–RNA polymerase interactions are governed by UvrD’s carboxy-terminal Tudor domain
Source: Commun Biol. 2020 Oct 23;3:607. doi: 10.1038/s42003-020-01332-2 (PMC7585439; doi:10.1038/s42003-020-01332-2)
Supplement: Supplementary file 1 — Supplementary Information [file 42003_2020_1332_MOESM1_ESM.docx]

**Supporting Information**

**for**

**UvrD helicase–RNA polymerase interactions are governed by UvrD’s carboxy-terminal Tudor domain.**

Ashish A. Kawale^1,2^, Björn M. Burmann^1,2,^*

^1^ Wallenberg Centre for Molecular and Translational Medicine, University of Gothenburg, Gothenburg, 40530, Sweden

^2^Department of Chemistry and Molecular Biology, University of Gothenburg, Gothenburg, 40530, Sweden

*Correspondence should be addressed to BMB: [bjorn.marcus.burmann@gu.se](mailto:bjorn.marcus.burmann@gu.se)

**Supplementary Figures:**

**
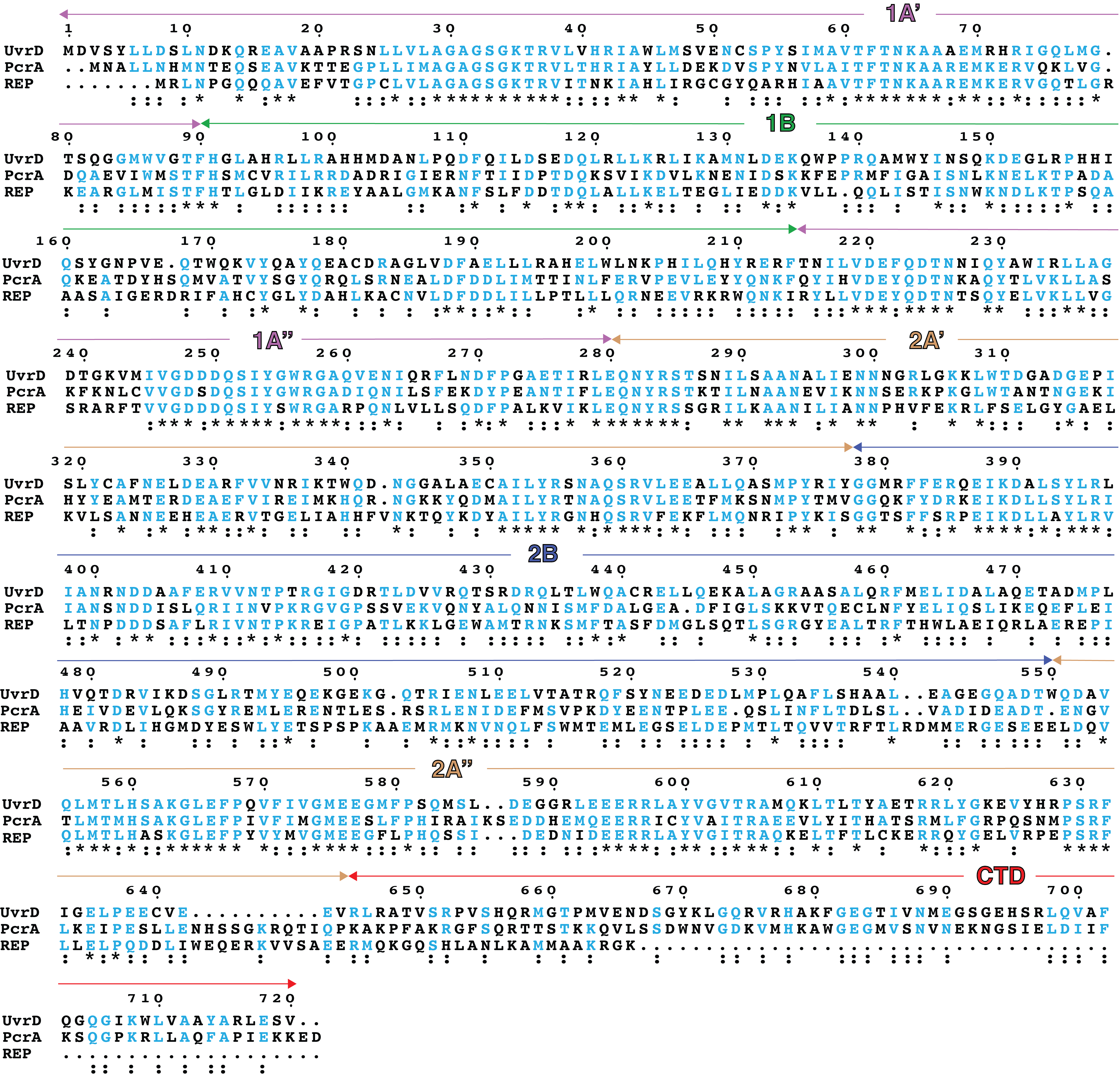
**

**Supplementary Figure 1: Sequence alignment of UvrD with its homologues PcrA and Rep helicases.** Sequences used in the analysis are of *E. coli* UvrD (1–720), of *S. aureus* PcrA (1–730), and of *E. coli* Rep helicase (1–673). Sequence alignment and analysis were performed by using Clustal Omega ^1^ and ESPript 3.0 ^2^ webservers, respectively. Sequence numbering and domain arrangement of UvrD (color code as in Figure 1) are indicated on the top of the alignment. Conserved residues are highlighted in cyan.

**
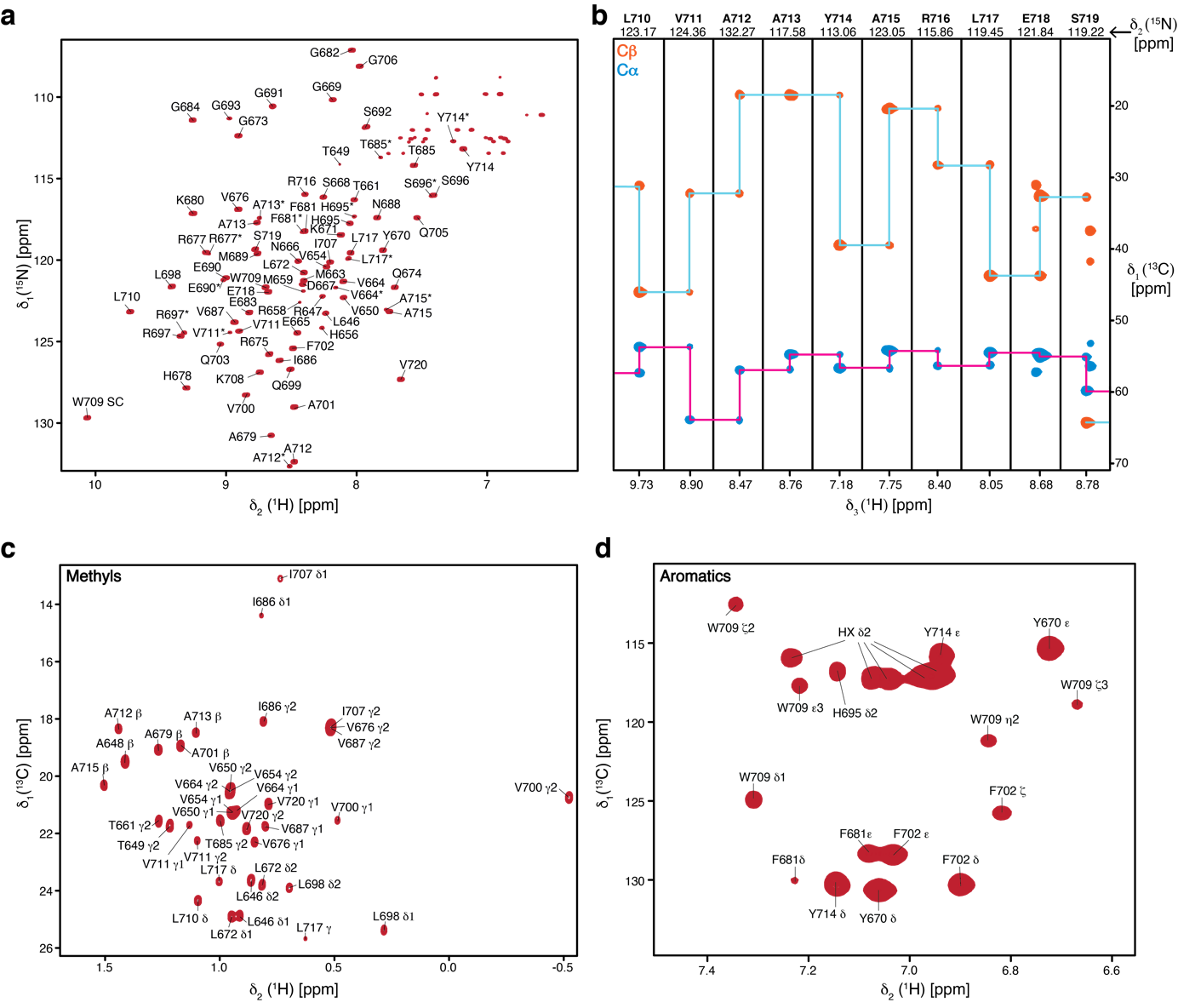
**

**Supplementary Figure 2: Sequence-specific resonance assignment of UvrD-CTD. a,** Annotated assignments of backbone signals on 2D [^15^N,^1^H]-NMR spectrum of [*U*-^13^C,^15^N] labeled UvrD-CTD (left panel). Resonances of the assigned peaks are marked with single-letter residue name and sequence number. **b,** Representative strips for residues (709–718) from a 3D HNCACB spectrum of UvrD-CTD. **c,** 2D [^13^C,^1^H]-NMR spectrum of [*U*-^13^C,^15^N] labeled UvrD-CTD annotated with assignments of methyl side-chain resonances. **d,** 2D [^13^C,^1^H]-NMR spectrum of [*U*-^13^C,^15^N] labeled UvrD-CTD annotated with assignments of aromatic side-chain resonances. Resonances belonging to the amino-terminal hexa-histidine-tag are denoted with HX δ2. All spectra were acquired in sample buffer (20 mM potassium-phosphate pH 6.5, 50 mM KCl) at 310K.


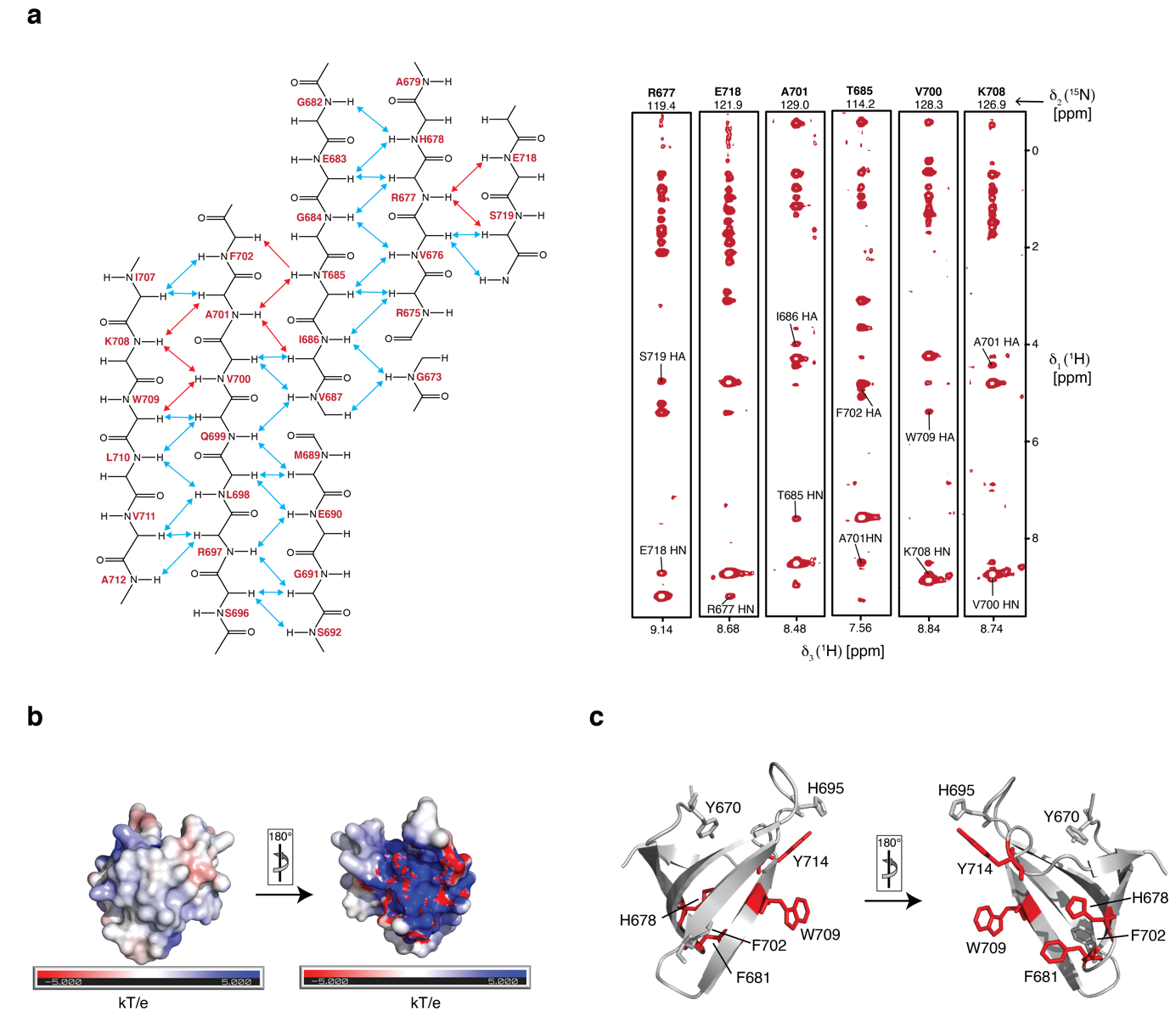


**Supplementary Figure 3: Structural details of the UvrD-CTD.** **a,** Schematic representation of the observed backbone inter-strand NOEs connecting UvrD-CTDs antiparallel β-strands (left panel). Representative NOE strips of a 3D ^13^C-NOESY showing inter-strand NOEs highlighted in red in the left panel (Right panel). **b**, Electrostatic surface representation of UvrD-CTD, generated using APBS plugin with positively- and negatively-charged surfaces represented in blue and red, respectively. **c**, Schematic representation of the aromatic residues of the UvrD-CTD possibly involved in aromatic cage formation mapped in red and sticks on the solution NMR structure in different orientations. Rest of the aromatic residues (except prolines) are depicted in grey.


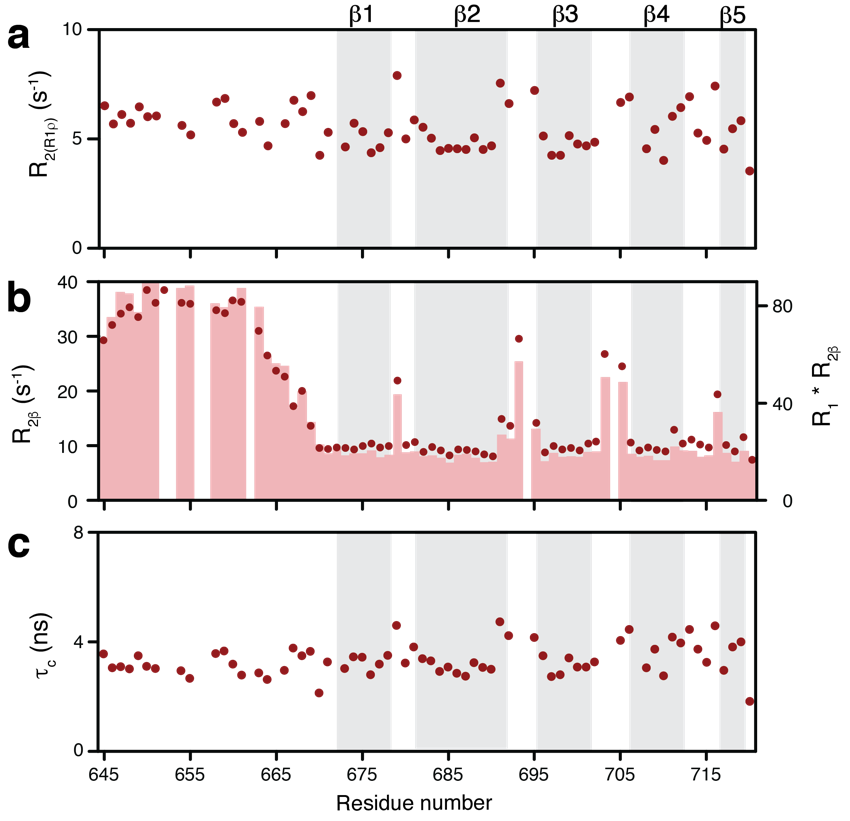


**Supplementary Figure 4: Assessing the slow timescale motions of UvrD-CTD. a,** ^15^N *R_2_* relaxation rates derived from the ^15^N *R*_1ρ_ rates report on the micro- to millisecond motions of UvrD-CTD. **b,** Slow time-scale (micro- to millisecond) motions of UvrD-CTD were also assessed by measuring *R*_2β_-rates (red circles) indicating exchange contributions of the amino-terminus and the loops connecting the β-strands. These values were consistent with the obtained *R*_1_ x *R*_2β_ values (bars). **c,** Rotational correlation time (τ_c_) plotted against residue number indicating the presence of six stable β-strands and an average τ_c_ of 3.64 ns for the structured region.

**
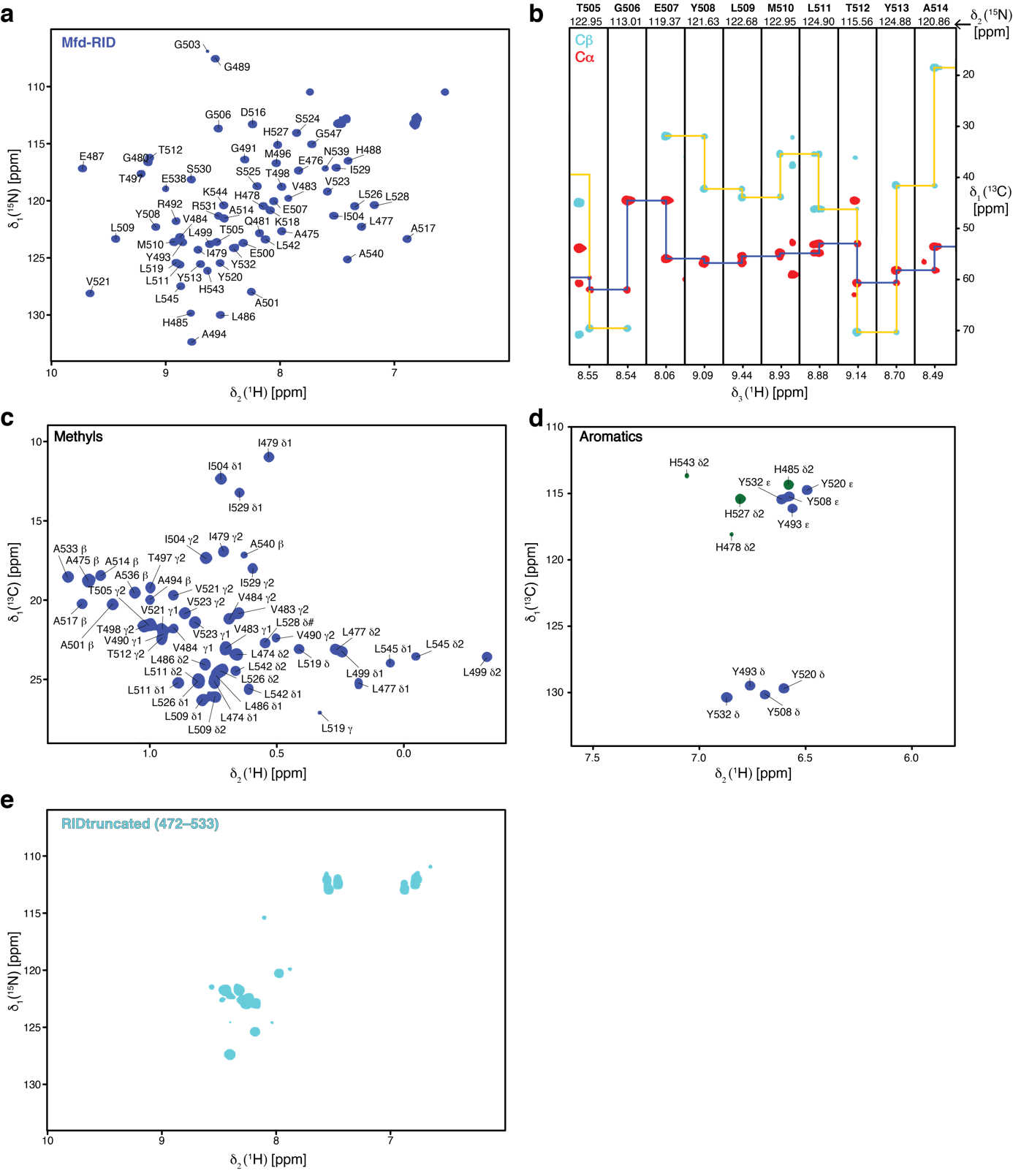
**

**Supplementary Figure 5: Resonance assignment of Mfd-RID. a,** Backbone resonance assignments annotated on 2D [^15^N,^1^H]-NMR spectrum of [*U*-^13^C,^15^N] labeled Mfd-RID. Single-letter residue name and sequence number are indicated on respectively assigned resonances. **b,** Sequence-specific resonance assignments depicted by representative strips for residues (505–514) from a 3D HNCACB spectrum of Mfd-RID. **c,** Methyl side-chain resonance assignment annotated on a 2D [^13^C,^1^H]-NMR spectrum of [*U*-^13^C,^15^N] labeled Mfd-RID. **d,** Aromatic side-chain resonance assignments annotated on a 2D [^13^C,^1^H]-NMR spectrum of [*U*-^13^C,^15^N] labeled Mfd-RID. **e,** 2D [^15^N,^1^H]-NMR spectrum of [*U*-^15^N] labeled truncated Mfd-RID (472–533) lacking the residues comprising β6 strand. All spectra were acquired in a PBS buffer pH 7.4 at 298 K.

**
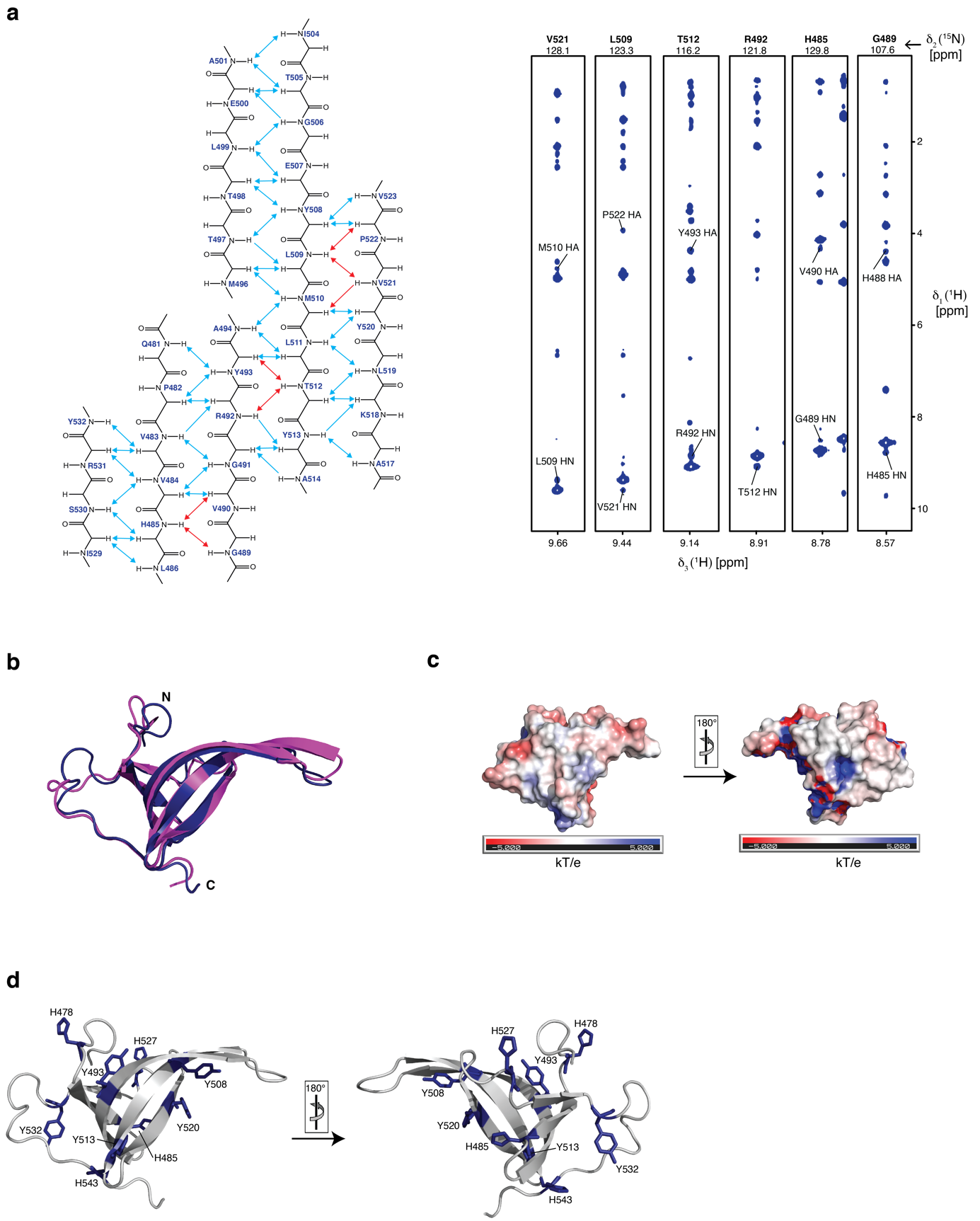
**

**Supplementary Figure 6: Mfd-RID structural features. a,** Scheme of the observed backbone inter-strand NOEs observed between Mfd-RIDs antiparallel β-strands (left panel). Representative NOE strips of a 3D ^13^C-NOESY showing inter-strand NOEs highlighted in red in the left panel (right panel). **b**, Structure alignment of Mfd-RID solution NMR structure (blue, this study) with the domain 4 (magenta) deduced from the previously reported Mfd-full length crystal structure (2EYQ) ^3^. **c,** Electrostatic surface representation of Mfd-RID, with positively- and negatively-charged surfaces represented in blue and red, respectively. **d,** Schematic representation of the aromatic residues of the Mfd-RID mapped on the solution NMR structure in different orientations.

**
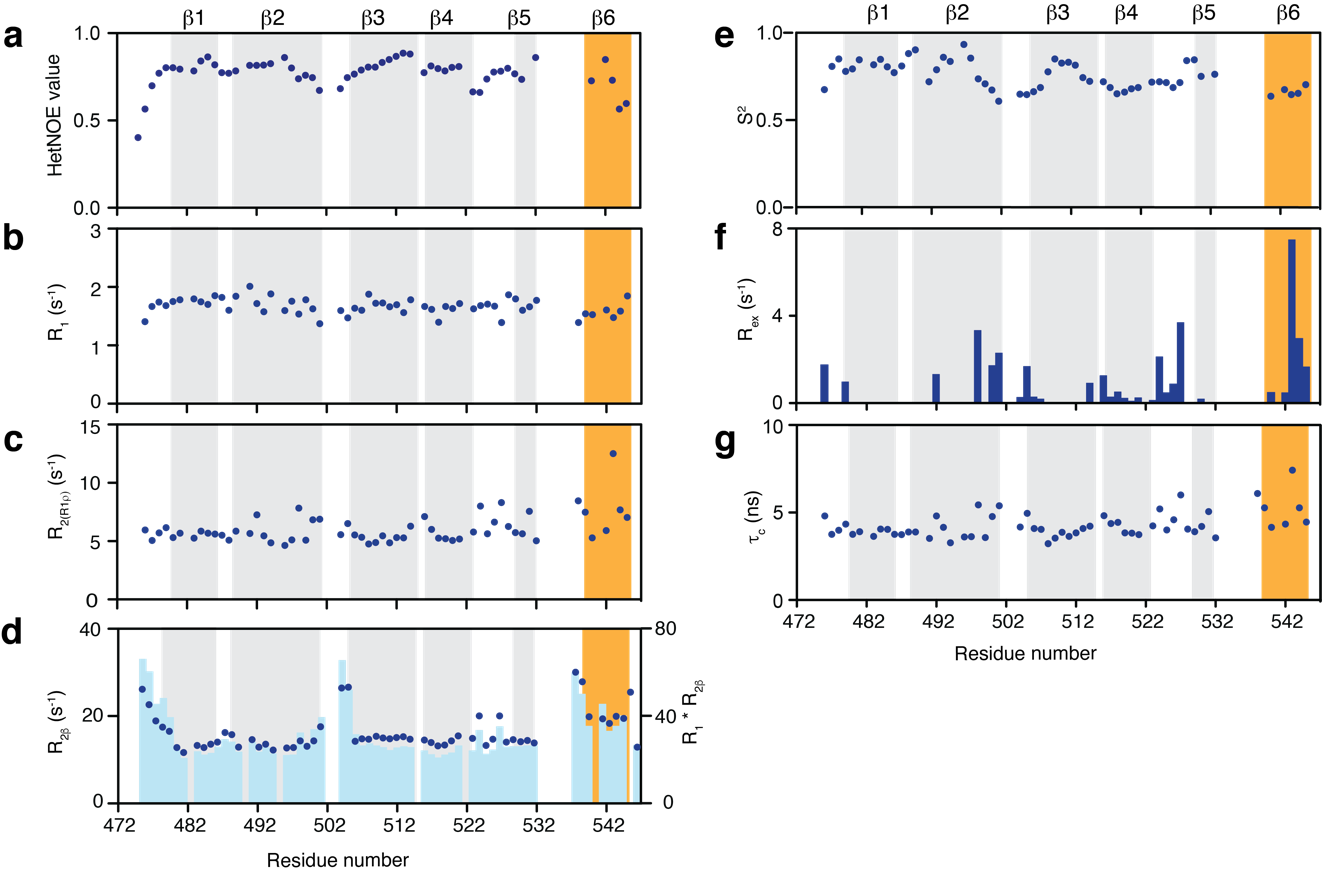
**

**Supplementary Figure 7: Backbone dynamics of Mfd-RID.** ^15^N-relaxation parameters for Mfd-RID, assessed by determining **(a)** ^15^N{^1^H}-NOE values, **(b)** *R*_1_, **(c)** *R*_2(R1ρ)_. **d,** Slow time-scale (μs–ms) motions of Mfd-RID were also assessed by measuring *R*_2β_-rates (blue circles) indicating exchange contributions of the amino-terminus and the loops connecting the β-strands. These values are consistent with the obtained *R*_1_ x *R*_2β_ values (bars). **e,f,** ^15^N NMR relaxation parameters were analyzed using the model-free formalism to determine generalized order parameters S^2^ (**e)** and the Chemical exchange rates *R*_ex_ **(f)** indicating motions on ps–ns and μs–ms timescales, respectively. **g,** Rotational correlation time (*τ*_c_) plotted against residue number indicating the presence of six stable β-strands and an average *τ*_c_ of 4.29 ns.


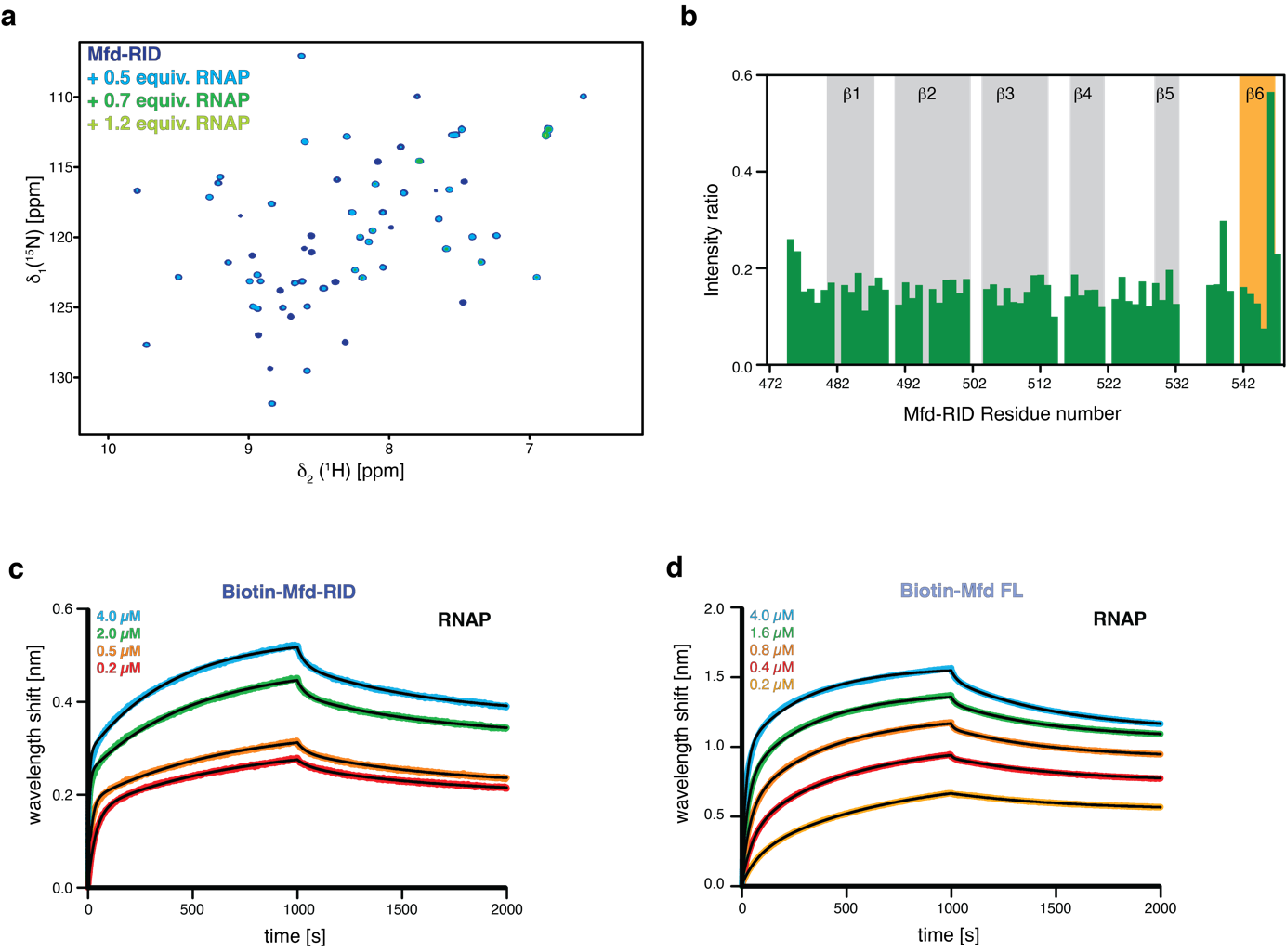


**Supplemental Figure 8: Investigation of the RNAP-binding capacity of Mfd-RID.** **a,** Overlay of 2D [^15^N,^1^H]-NMR spectra of [*U*-^15^N] Mfd-RID in the absence (blue) and in the presence of increasing amounts of RNA polymerase (as indicated) acquired in the PBS buffer at 310K. **b,** The ratio of individual peak intensities in the presence of 0.7 molar excess of RNA polymerase (core enzyme) to the apo-Mfd-RID, plotted against residue numbers. β1–β5 strands are highlighted in the grey background whereas β6 is marked with the orange background. **c, d,** Biolayer interferometry (BLI) data analysis of Mfd-RID **(c)** and Mfd full-length **(d)** binding to RNAP (core enzyme), respectively. Analyte concentrations are indicated in each figure. Non-linear least-square fits to the experimental data are indicated by the black lines.


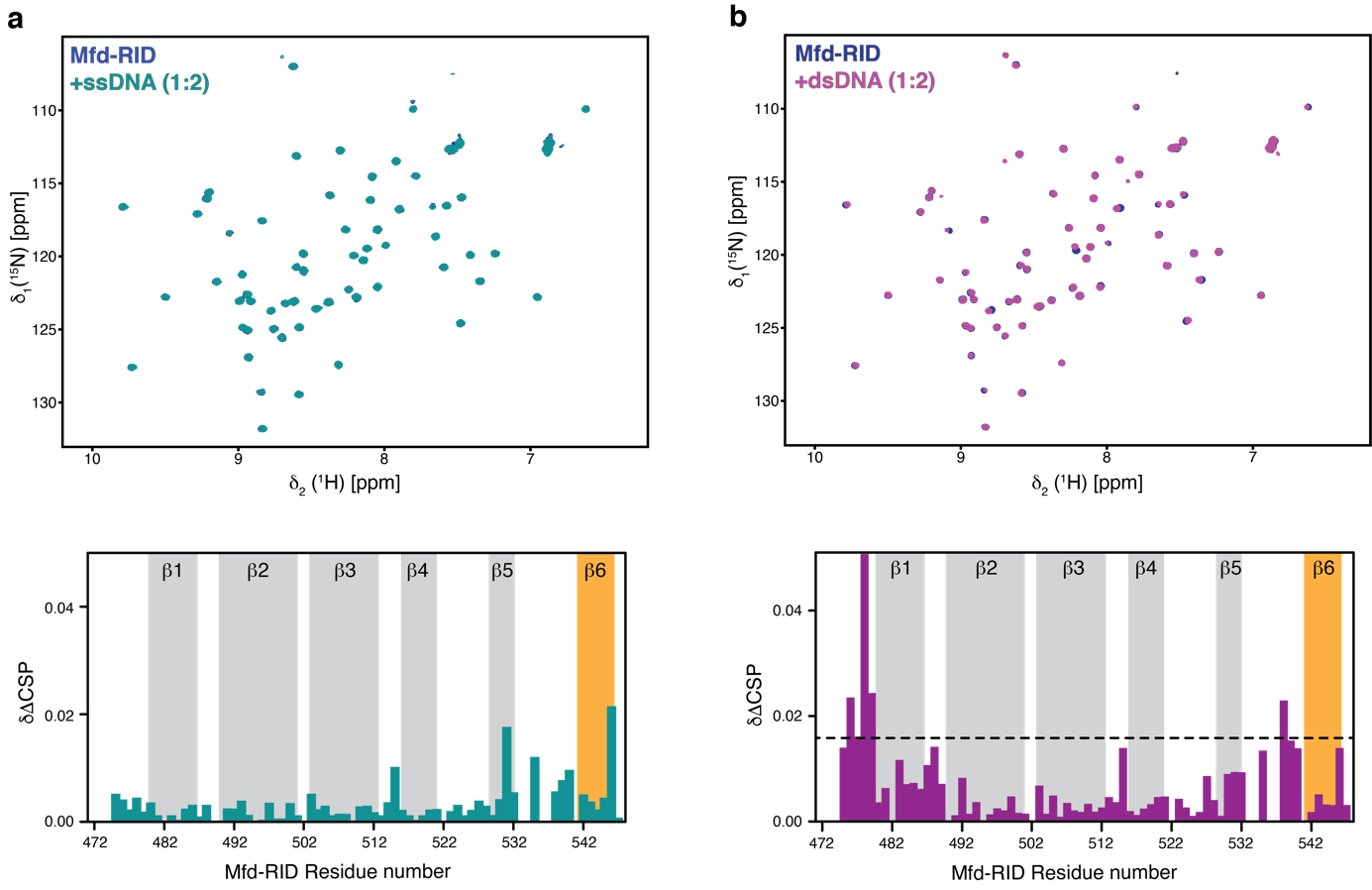


**Supplementary Figure 9: Mfd-RID shows no specific DNA binding. a,** Overlay of 2D [^15^N,^1^H]-NMR spectra of [*U*-^15^N] Mfd-RID in the absence (blue) and in the presence (green) of two molar excess of 17mer (ssDNA). Chemical shift perturbation versus residue numbers of Mfd-RID plotted in the presence of two molar equivalent of ssDNA (bottom). **b,** Overlay of 2D [^15^N,^1^H]-NMR spectra of [*U*-^15^N] Mfd-RID in the absence (blue) and in the presence (magenta) of two molar excess of 35mer (dsDNA). Chemical shift perturbation versus residue numbers of Mfd-RID plotted in the presence of two molar equivalent of dsDNA (bottom). Secondary structural elements are highlighted. Black dotted line represents the twice the standard deviation. Both NMR titrations were performed in PBS buffer at 298K.

**
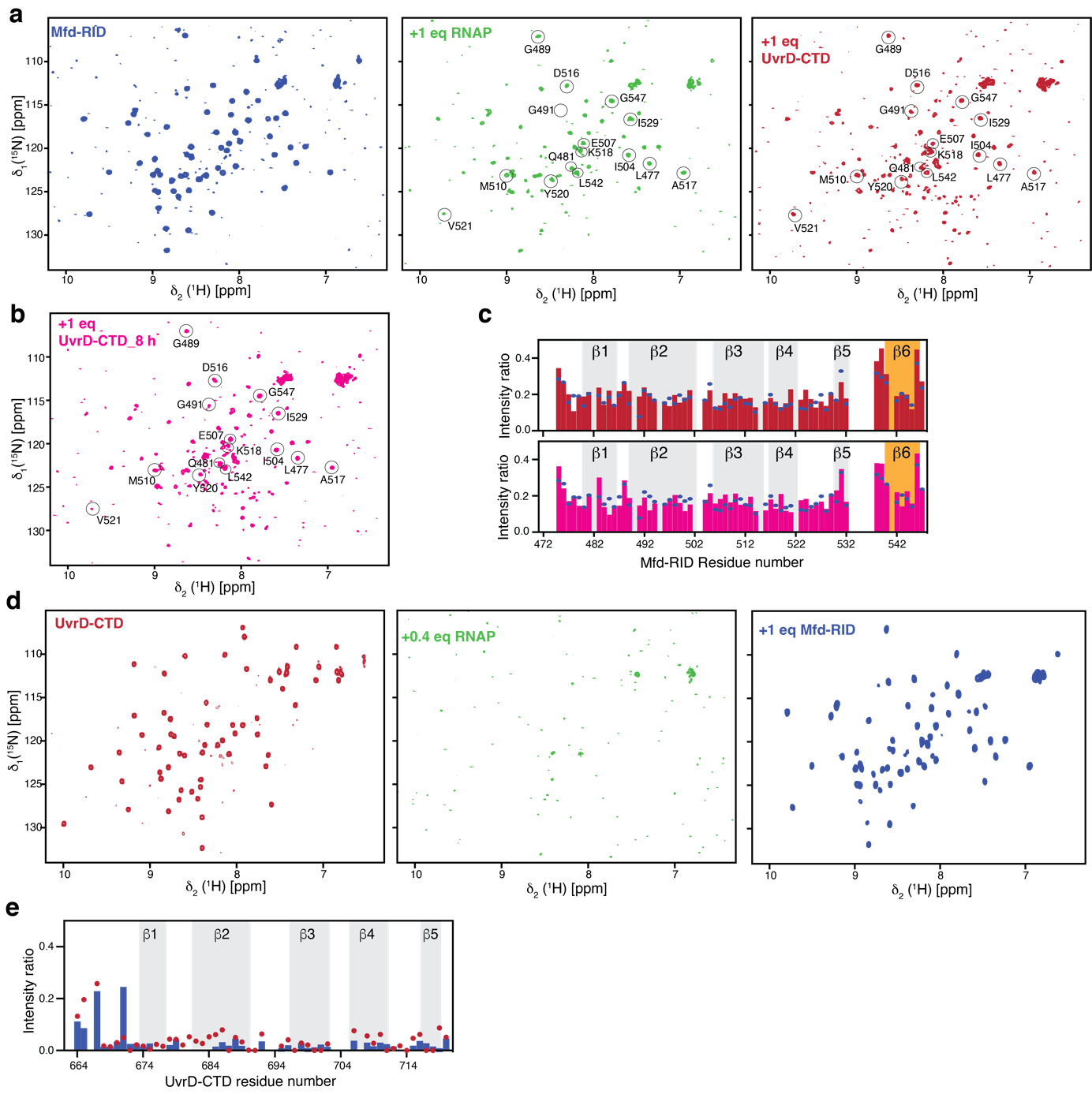
**

**Supplementary Figure 10: UvrD-CTD and Mfd-RID do not compete for RNAP binding. a,** 2D [^15^N,^1^H]-NMR spectrum of [*U*-^15^N] Mfd-RID in the absence (left panel), upon addition (middle panel) of one molar equivalent of RNAP and further addition of one equivalent of [*U*-^15^N] UvrD-CTD (right panel), respectively. Mfd-RID resonances slightly gaining intensity after addition of equimolar UvrD-CTD to the Mfd-RID in the presence of equimolar RNAP, are encircled and annotated. **b,** 2D [^15^N,^1^H]-NMR spectrum of [*U*-^15^N] Mfd-RID, upon addition of equimolar RNAP and further addition of one equivalent of [*U*-^15^N] UvrD-CTD after approximately eight hours of incubation at 298 K. **c,** The ratio of Mfd-RID peak intensities in the presence of equimolar RNAP (blue dots) and in the presence of equimolar RNAP and UvrD-CTD (red bars) to the apo-Mfd-RID plotted against residues numbers (top panel). The ratio of Mfd-RID peak intensities in the presence of equimolar RNAP (blue dots) and in the presence of equimolar RNAP and UvrD-CTD (magenta bars) after incubation at 298 K for approximately eight hours to the apo-Mfd-RID plotted against residues numbers (top panel). **d,** 2D [^15^N,^1^H]-NMR spectrum of [*U*-^15^N] UvrD-CTD in the absence (left panel), upon addition (middle panel) of 0.4 molar equivalent of RNAP and further addition of one molar equivalent of [*U*-^15^N] Mfd-RID (right panel). **e,** The ratio of UvrD-CTD peak intensities in the presence of 0.4 molar equivalent RNAP (red dots) and in the presence of 0.4 molar equivalent RNAP and equimolar Mfd-RID (blue bars) to the apo-UvrD-CTD plotted against residues numbers.

**
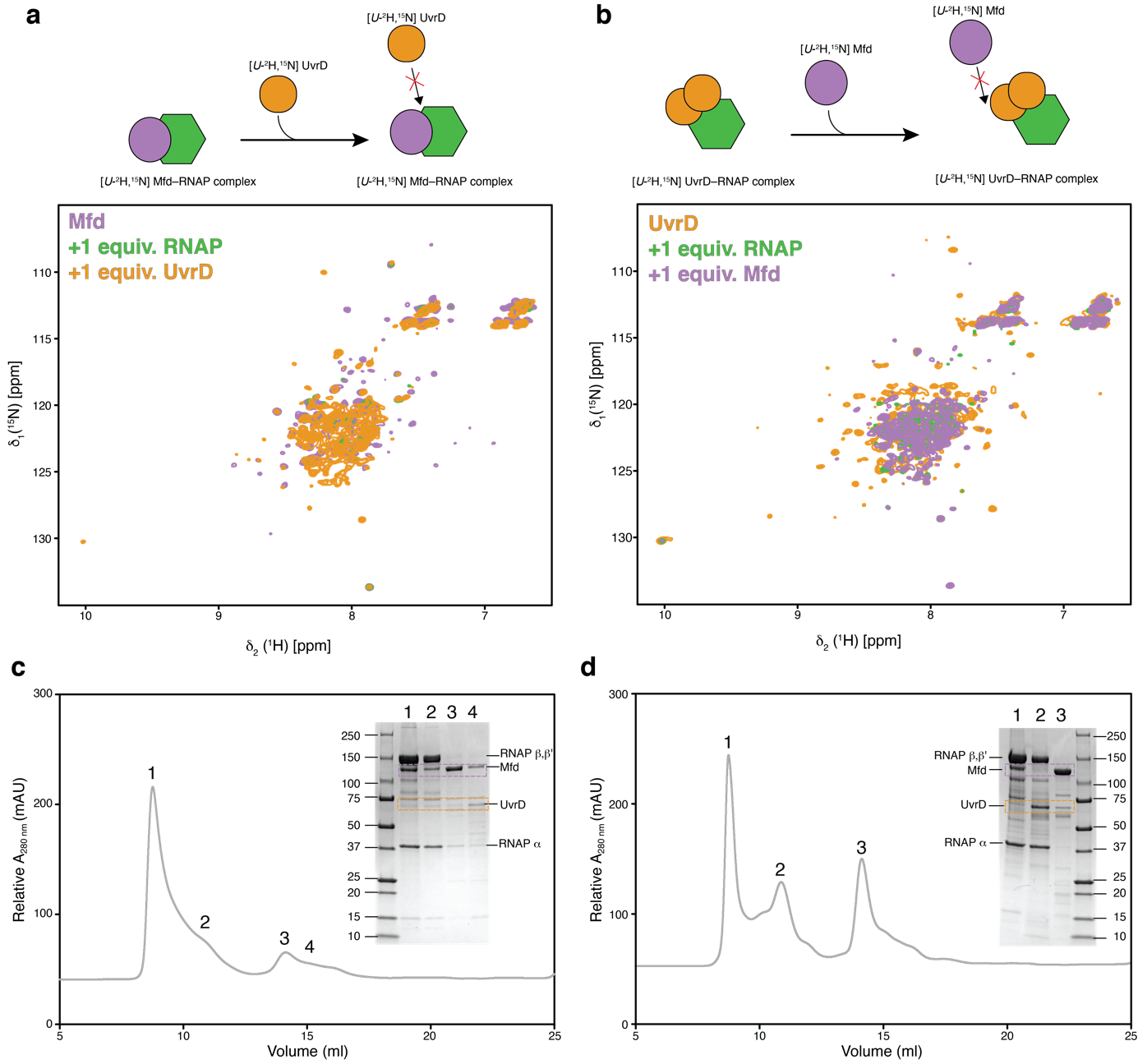
**

**Supplementary Figure 11: UvrD and Mfd do not dissociate each other from the RNAP complex. a,** Illustration of the experimental setup (top panel). Overlay of 2D [^15^N,^1^H]-NMR spectra of [*U*-^2^H,^15^N] Mfd in the absence and in the presence of one molar equivalent of RNAP and [*U*-^2^H,^15^N] UvrD, respectively (bottom panel). **b,** Experimental design (top panel) and overlay of 2D [^15^N,^1^H]-NMR spectra of [*U*-^2^H^15^N] UvrD in the absence and in the presence of one molar equivalents of RNAP and one molar equivalent of [*U*-^2^H^15^N] Mfd (bottom panel). Both NMR titrations were performed in PBS buffer at 298K. **c,** Size exclusion chromatography elution profile for the samples after the displacement experiment shown in in panel **a**. **d,** Size exclusion chromatography elution profile for the samples after the displacement experiment shown in panel **b**.

**
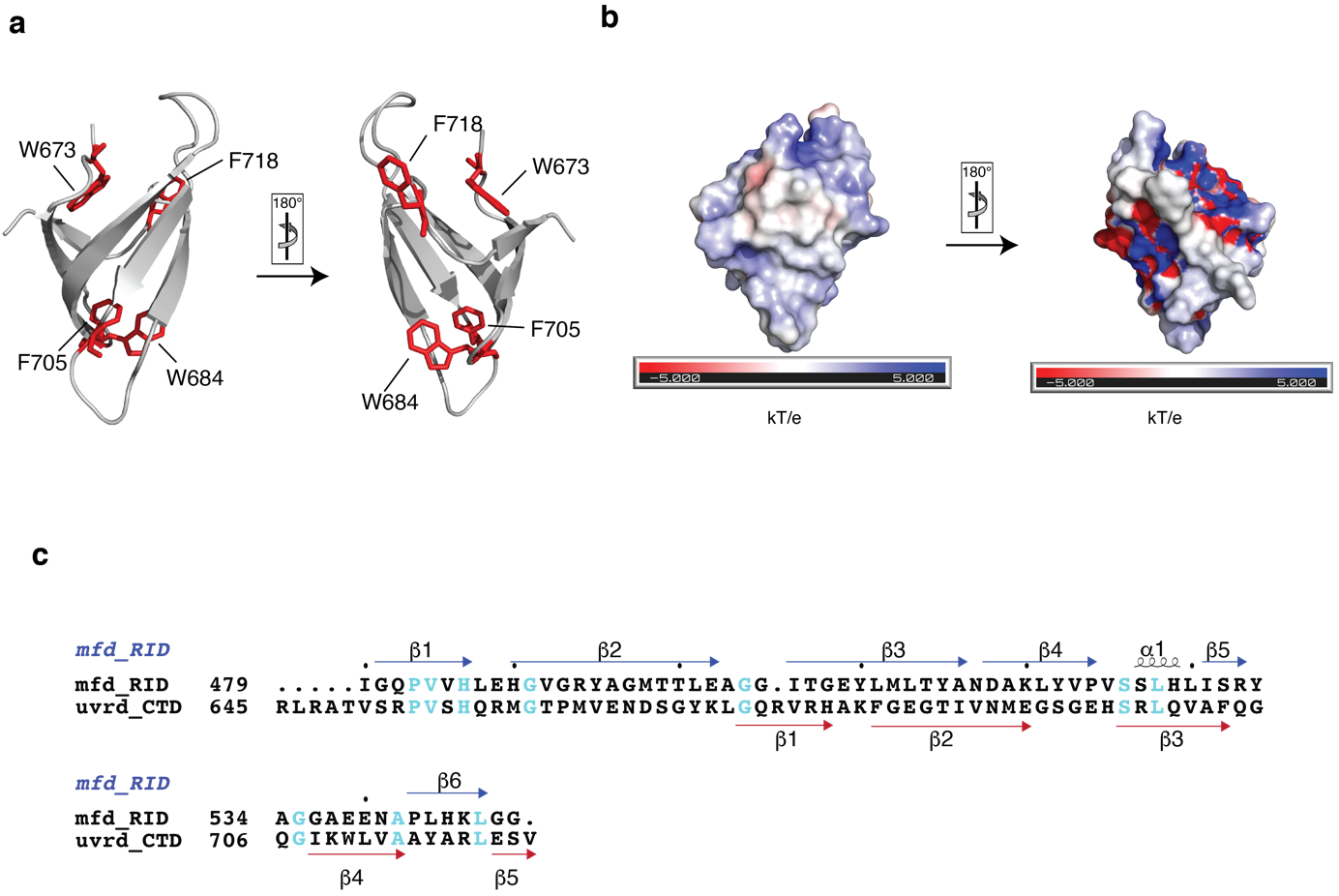
**

**Supplementary Figure 12: Structural comparison of PcrA and UvrD as well as primary sequence comparison of *E. coli* Mfd-RID and UvrD-CTD. a,** Schematic representation of the aromatic residues of the PcrA-CTD possibly involved in aromatic cage formation mapped in red and sticks on the crystal structure (PDB ID: 5DMA) ^4^ in different orientations. **b,** Electrostatic surface representation of PcrA-CTD, with positively- and negatively-charged surfaces represented in blue and red, respectively. **c,** Mfd-RID (474–547) and UvrD-CTD (645–720) amino acid sequences from *E. coli* aligned using Clustal Omega ^1^ and analyzed using ESPript 3.0 ^2^ webserver. Sequence numbering and secondary structural elements of Mfd-RID and UvrD-CTD are shown in blue and red color respectively. Conserved residues are highlighted in cyan.

**Supplementary Tables:**

**Supplementary Table 1:** Dissociation constants (*K*_D_) derived for different UvrD and Mfd constructs with RNAP

| **Binding pair** | ***K*_D_ (nM)** | **R^2^** | **ζ^2^** |
| --- | --- | --- | --- |
| UvrD FL–RNAP | 1000 ± 130 | 0.9864 | 0.0002 |
| UvrD ΔCTD–RNAP | 1900 ± 150 | 0.9957 | 0.0002 |
| UvrD-CTD–RNAP | 77 ± 2.8 | 0.9965 | 0.0001 |
| Mfd FL–RNAP | 310 ± 14 | 0.9965 | 0.0017 |
| Mfd RID–RNAP | 390 ± 42 | 0.9810 | 0.0005 |

**Supplementary Table 2:** Dissociation constants (*K*_D_) derived for UvrD-CTD with DNA

| **Binding pair** | ***K*_D_ (µM)** | **R^2^** | **ζ^2^** |
| --- | --- | --- | --- |
| ssDNA–UvrD-CTD | 3.8 ± 0.47 | 0.9948 | 0.0017 |
| dsDNA–UvrD-CTD | 1.9 ± 0.17 | 0.9969 | 0.0006 |

**Supplementary Table 3:** Overview of the plasmids used in this study.

| UVRD_FL | pET-28d (+) with uncleavable N-terminal hexahistidine tag | Epshtein *et al.* ^5^ |
| --- | --- | --- |
| UVRD_CTD | pET-28d (+) with uncleavable N-terminal hexahistidine tag | This study |
| UVRD_ΔCTD | pET-28d (+) with uncleavable N-terminal hexahistidine tag | This study |
| MFD_FL | pET-28b (+) with N-terminal hexahistidine with TEV cleavage site | This study |
| MFD_RID | pET-28b (+) with N-terminal cleavable Sumo tag | This study |
| RNAP_pIA900 | pMB1 expressing α–β–β’–ω subunits, containing C-terminally decahistidine tagged β’ with TEV cleavage site | Svetlov *et al.* ^6^ |

**Supplementary Table 4:** Overview of the primers used in this study.

| UVRD_FL_FWD | 5’-CAT GCC ATG GAA TGG ACG TTT CTT ACC TGC TC-3’ |
| --- | --- |
| UVRD_FL_REV | 5’-GAT CCT CGA GTT ACA CCG ACT CCA GCC GGG C-3’ |
| UVRD_CTD_FWD | 5’-CAT GCC ATG GAC GCC TGC GCG CCA CGG-3’ |
| UVRD_CTD_REV | 5’-GAT CCT CGA GTT ACA CCG ACT CCA GCC GGG C-3’ |
| UVRD_ΔCTD_FWD | 5’-ACG CCG ATG GTC TAG AAC GAC AGC GGC-3’ |
| UVRD_ΔCTD_REV | 5’-GCC GCT GTC GTT CTA GAC CAT CGG CGT-3’ |
| MFD_RID_FWD | 5’-CGC ATC TGG AAC AGA TTG GCG GTC GTA AC CTT GCG GAA CTG CAT-3’ |
| MFD_RID_REV | 5’-GTG GTG GTG GTG GTG CTC GAG TTA GCC GCC AAG TTT ATG CAG CG-3’ |

**Supplementary References:**

1. Sievers F.*, et al.* Fast, scalable generation of high-quality protein multiple sequence alignments using Clustal Omega. *Mol. Syst. Biol.* **7**, 539 (2011).

2. Robert X., Gouet P. Deciphering key features in protein structures with the new ENDscript server. *Nucleic Acids Res.* **42**, W320-324 (2014).

3. Deaconescu A.M.*, et al.* Structural basis for bacterial transcription-coupled DNA repair. *Cell* **124**, 507–520 (2006).

4. Sanders K.*, et al.* The structure and function of an RNA polymerase interaction domain in the PcrA/UvrD helicase. *Nucleic Acids Res.* **45**, 3875–3887 (2017).

5. Epshtein V.*, et al.* UvrD facilitates DNA repair by pulling RNA polymerase backwards. *Nature* **505**, 372–377 (2014).

6. Svetlov V., Artsimovitch I. Purification of bacterial RNA polymerase: tools and protocols. *Methods Mol. Biol.*, 13–29 (2015).
